# *In vivo* Evidence for Partial Activation of Eosinophils via the Histamine H_4_-Receptor: Adoptive Transfer Experiments Using Eosinophils From H_4_R^−/−^ and H_4_R^+/+^ Mice

**DOI:** 10.3389/fimmu.2018.02119

**Published:** 2018-09-25

**Authors:** Bastian Schirmer, Luisa Bringmann, Roland Seifert, Detlef Neumann

**Affiliations:** Institute of Pharmacology, Hannover Medical School, Hannover, Germany

**Keywords:** experimental colitis, histamine H_4_ receptor, histamine, cytokines, inflammatory bowel diseases

## Abstract

Our previous *in vitro* studies revealed that histamine via histamine the H_4_-receptors (H_4_R), as compared to other stimuli, such as eotaxin or formylpeptides, rather partially activates eosinophilic granulocytes (eosinophils). In order to evaluate the H_4_R-mediated activation of eosinophils *in vivo*, we employed dextran sodium sulfate (DSS)-induced colitis in mice, closely resembling human ulcerative colitis (UC), which is largely characterized by a local eosinophilic infiltration of the colon. IL-5-deficient BALB/c mice served as a model with reduced endogenous numbers of eosinophils, in which wild-type (H_4_R^+/+^) or H_4_R-deficient (H_4_R^−/−^) eosinophils were adoptively transferred during the course of DSS-induced colitis. During the 1-week observation period, transfer of eosinophils transiently reversed the acute clinical colitis-like phenotype (body weight loss, perianal bleeding, soft stool consistency) resulting from IL-5-deficiency. This reversion was significantly more pronounced upon transfer of eosinophils from H_4_R^+/+^ mice as compared to those from H_4_R^−/−^ mice. Already at the end of the observation period, the clinical effects of the transfer of H_4_R^+/+^ and H_4_R^−/−^ eosinophils became similar, as were the results of the histological examination of the cola and the analyses of cytokine production in cola and in re-stimulated lymph node cells performed at this time. Thus, analyzing clinical and pathological parameters representative of colitis in this model, we demonstrate that as well as *in vitro*, also *in vivo* histamine via the H_4_R only partially activates eosinophils.

## Introduction

Histamine [2-(4-imidazolyl)-ethylamine] is involved in a broad variety of (patho)-physiological processes, such as the regulation of gastric acid production, neurotransmission, and allergic and other inflammatory responses. To selectively exert these diverse functions, histamine binds to specific receptors of which four subtypes demonstrating selective expression patterns have been identified. These histamine receptors all belong to the class of G protein-coupled receptors and are referred to as H_1_-receptor (H_1_R), H_2_R, H_3_R and H_4_R (1). While the effects of histamine on gastric acid production and neurotransmission are mediated by H_2_R and H_3_R, respectively, the regulation of inflammatory responses by histamine involves H_1_R and H_4_R (2, 3).

Eosinophils functionally express the H_4_R (4–8). In primary human eosinophils, the *in vitro* ligand-mediated activation of H_4_R induces intracellular calcium ion mobilization and cell migration, but not reactive oxygen species production and degranulation. Moreover, calcium mobilization and migration are induced more effectively by e.g., eotaxin and fMLP, which also induce reactive oxygen species production and degranulation. Thus, in an *in vitro* setting, H_4_R mediates a rather partial activation of eosinophils (8). Whether this holds true also *in vivo* has not been addressed so far.

Elevated numbers of eosinophils and high histamine concentrations are found in inflamed colon tissues of patients suffering from inflammatory bowel diseases (IBD), which are idiopathic, chronic-recurring diseases of the gastrointestinal system with the two major clinical manifestations ulcerative colitis (UC) and Crohn's disease (CD) (9–11), as well as in cola of mice subjected to chemically induced colitis models (12, 13). While antagonists at the H_1_R are without clinical relevance in the treatment of IBD or in animal models, blockade of histamine production or H_4_R function is beneficial in specific colitis models, such as dextran sodium sulfate (DSS)-induced colitis in mice (14–18). Thus, DSS-induced colitis in mice provides a good and reliable model to study the effect of H_4_R on eosinophil function *in vivo*.

IL-5 also contributes to this process. Although recruitment of eosinophils to inflamed tissue is largely initiated by eotaxin, IL-5 contributes to this process, too (19, 20), and IL-5-deficient mice show a significant reduction in the overall numbers of eosinophils and in the prevalence of colon-infiltrating eosinophils during DSS-induced colitis (13).

Hence, in this study we adoptively transferred eosinophils from H_4_R-deficient (H_4_R^−/−^) and H_4_R-competent, i.e. wild-type (H_4_R^+/+^) mice into IL-5-deficient BALB/cJ mice and thereafter subjected these reconstituted mice to DSS-induced colitis. We demonstrate that IL-5 deficiency in BALB/cJ mice is protective against DSS-induced colitis and that adoptive transfer of eosinophils into these mice transiently reverts the acute colitis phenotype, eosinophils from H_4_R^+/+^ mice being more efficient than those from H_4_R^−/−^ mice. Moreover, the H_4_R on eosinophils promotes shifting the balance of the beginning local immune response toward the Th2 type (13).

## Materials and methods

### Materials

All chemicals and reagents used had been purchased from Sigma-Aldrich Chemie GmbH (Munich, Germany), if not stated otherwise.

### Animals

Animal housing and experimental procedures were approved by the animal welfare committee of the Hannover Medical School, complied with the German animal welfare legislation and were finally approved by the Lower Saxony State Office for Consumer Protection and Food Safety (LAVES, AZ 33.12-42502-04-12/1021). All mice were housed according to directive 2010/63/EU under specific pathogen-free conditions at the central animal facility of the Hannover Medical School and fed with standard diet (Altromin 1320, Altromin Spezialfutter GmbH & Co. KG, Lage, Germany). C57BL/6-Il5tm1Kopf/J mice (21) were purchased from Charles River Deutschland (Sulzbach, Germany) and backcrossed for at least ten generations onto BALB/cJRj mice purchased from Janvier Labs (Le Genest-Saint-Isle, France). Since then, the IL-5-deficient mice were routinely maintained as homozygous breeding. H_4_R^−/−^ mice (strain: C.129HrH${}_{4}^{\text{tm}1\text{Lex}}$) were generated by Lexicon Genetics (Woodlands, TX, USA) (22) and backcrossed for at least 10 generations onto the BALB/cJRj strain. Both H_4_R^+/+^ and H_4_R^−/−^ mice were bred from heterozygous matings of these founder animals. 8–10 weeks old male mice were used for the experiments. Littermates were chosen as control mice.

### Generation of bone-marrow derived eosinophilic granulocytes (BM-Eos)

BM-Eos were generated as previously described (23). Briefly, bone marrow was flushed from femorae and tibiae of donor mice (either H_4_R^+/+^ or H_4_R^−/−^ mice) with RPMI-1640 + 10% (v/v) Fetal Calf Serum (FCS), 2 mM EDTA. Erythrocytes were lysed by adding ice-cold 0.2% (w/v) NaCl in water to the pelleted cells. After 20 s, lysis was stopped by adding an equal volume of 1.6% (w/v) NaCl. Bone marrow cells were then cultured at 37°C, 5% (v/v) CO_2_ at 1 × 10^6^ cells/ mL in base medium [RPMI-1640 + 20% (v/v) FCS, 25 mM HEPES, 100 IU/ml penicillin, 10 μg/ml streptomycin, 2 mM glutamine, 1 × NEAA (non-essential amino acids), 1 mM sodium pyruvate, 50 μM ß-Mercaptoethanol (β-MeSH)] + 100 ng/ml SCF, 100 ng/ml Flt3-L (both PeproTech GmbH, Hamburg, Germany) for 4 days. From day 4 on, the cells were cultured in base medium + 40 ng/ml IL-5 (Miltenyi Biotec GmbH, Bergisch Gladbach, Germany). Cells were used between 10 and 12 days after initiation of the culture and were routinely >90% eosinophils as detected by flow cytometric evaluation of Siglec-F expression (Anti-Siglec-F, mouse, clone: ES22-10D8; Miltenyi Biotec GmbH, Bergisch Gladbach, Germany).

### Induction of colitis by DSS, adoptive transfer of BM-Eos and animal dissection

**Figure 1:**
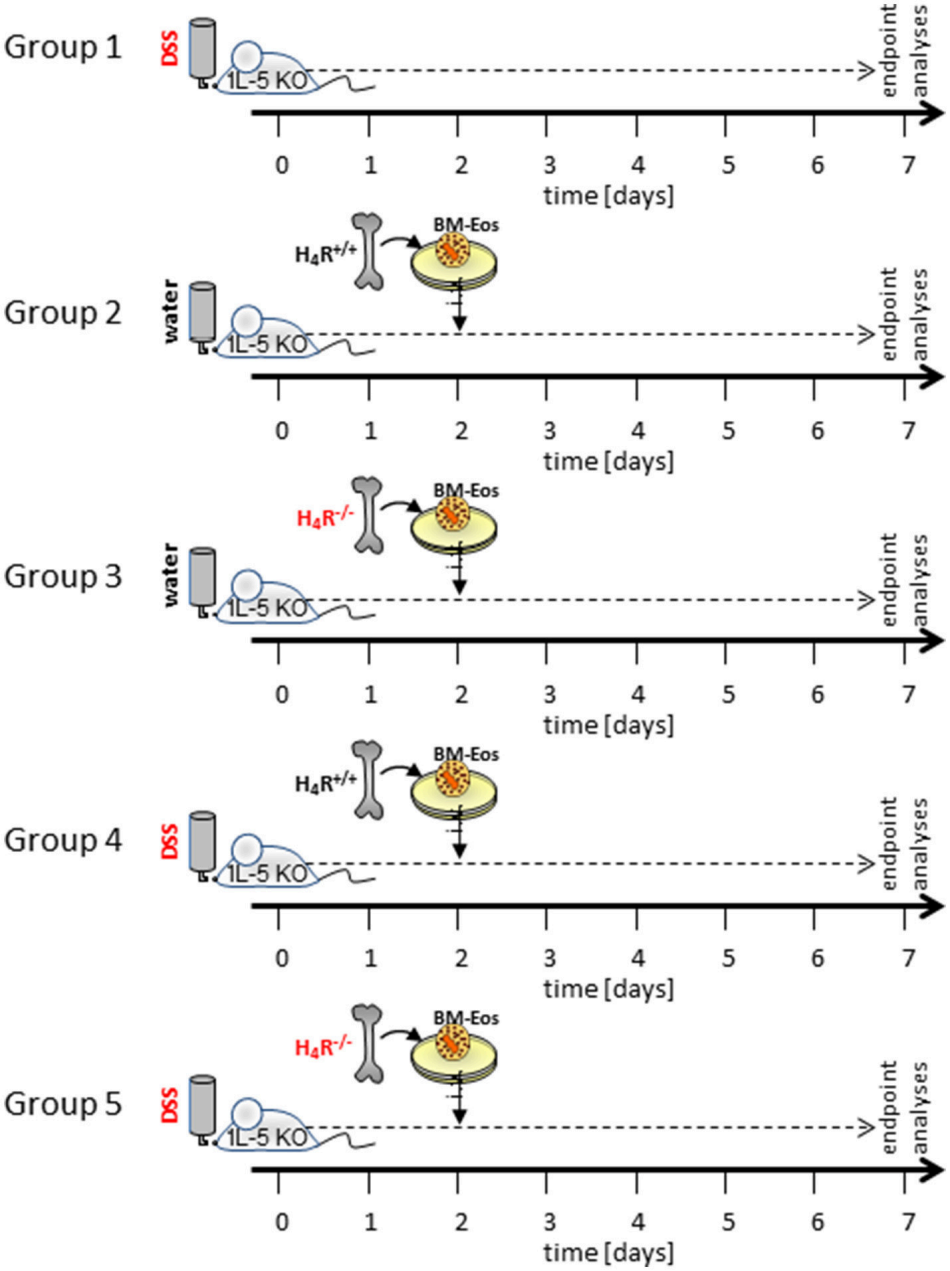
Schematic representation of the experimental settings. Not shown are the control settings water- and DSS-fed WT mice and water-fed IL-5-deficient mice without reconstitution of eosinophils.

Acute colitis was induced at day 0 by adding 3% (w/v) DSS (36,000–50,000 Da; MP Biomedicals, Santa Ana, CA, USA) to their drinking water. DSS was fed to the mice of the colitis groups from day 0 to day 7. Water-fed mice served as control. On day 2, BM-Eos were suspended in RPMI-1640 at 1 × 10^7^ cells/ ml and 200 μL of this cell suspension per mouse (= 2 × 10^6^ cells) were injected intravenously into the tail veins of IL-5-deficient mice (Figure 1). WT or IL-5-deficient mice without i.v. injection of cells served as DSS-fed controls. On day 7, mice were sacrificed by exsanguination after sedation with CO_2_, the sera were collected and the cola and ceca were removed. The lengths of cola and ceca were measured and the collected tissues were washed with PBS to remove remaining feces. One third of each colon was fixed in 4% (v/v) formaldehyde in PBS, embedded in paraffin, and further processed for hematoxylin/eosin (H&E) staining. Another third of each colon was stored in RNAlater for qPCR analyses, the remaining third of each colon was snap frozen in 2 mL Matrix D FastPrep® tubes (MP Biomedicals, Santa Ana, CA, USA). Mesenteric lymph nodes were removed and a lymphocyte enriched single cell suspension was generated by passing the tissue through a sterile 100 μm mesh with 5 mL RMPI-1640 medium.

### Generation of colon homogenates and supernatants of mesenteric lymph nodes

Mesenteric lymph node cells were seeded at 1 × 10^6^ cells/ml 200 μl/well in RPMI-1640 + 10% (v/v) FCS, 50 μM ß-MeSH on a 96 well-plate, and incubated at 37°C, 5% (v/ v) CO_2_ for 24 h in the absence or presence of CD3-antibody (EXBIO Praha, Vestec u Prahy, Czech Republic). After the incubation period, cell-free supernatants were generated by centrifugation at 300 g for 10 min at 4°C. Colon tissues were homogenized either for cytokine measurements in ice cold PBS + 1x protease inhibitor cocktail (Cell Signaling Technology, Frankfurt, Germany) or for RNA extraction in lysis buffer RA1 (Macherey Nagel, Düren, Germany) supplemented with 150 mM ß-MeSH using the FastPrep® 24 homogenizer and 2 mL Matrix D FastPrep® tubes (both from MP Biomedicals, Santa Ana, CA, USA) according to the manufacturer's instructions for intestinal tissue.

### Cytokine measurements

Concentrations of Ccl2, Cxcl1, Cxcl2, interleukin (IL)-1β, IL-5, IL-6, IL-10, IL-12p70, IL-17A, IL-23p19, interferon (IFN)-γ, and TNF were measured in colon homogenates, sera, and cell-free supernatants of αCD3-stimulated lymph node cells with a customized multiplex magnetic Luminex Kit (R&D Systems, Minneapolis, MN, USA). Myeloperoxidase concentrations were evaluated using a commercially available DuoSet ELISA Kit (R&D Systems, Minneapolis, MN, USA). Cytokine measurements in colon tissue were normalized to the total protein content of the homogenates.

### Quantitative real-time PCR

Total RNA was extracted from colon tissue stored in RNAlater with NucleoSpin® RNA II kit (Macherey Nagel, Düren, Germany) according to the manufacturer's instruction. cDNA was prepared from 1 μg of total RNA by reverse transcription for 30 min at 50°C with Maxima Reverse Transcriptase (Thermo Fisher Scientific, Waltham, MA, USA). Sequences specific for the transcription factors Tbet1 (Assay ID: Mm00450960_m1) and Gata3 (Mm00484683_m1) were quantified in relation to Gapdh (Mm99999915_g1) as reference gene using TaqMan™ assays (Thermo Fisher Scientific, Waltham, MA, USA).

### Evaluation of disease activity

The disease severity of colitic mice was assessed daily by calculating a disease activity index (DAI) ranging from 0 to 12. The DAI is based on total body weight loss (0: no weight loss, 1: 1–5%, 2: 5–10%, 3: 10–15%, 4: >15%), stool consistency (0: normal, 2: soft, 4: diarrhea) and rectal bleeding (0: no bleeding, 2: little bleeding, 4: massive bleeding).

### Histological analysis

H/E-stained tissue slices of colon segments were analyzed in a blinded fashion by two independent researchers. A modified histological severity score (24) was calculated by evaluating overall severity of inflammation (0: normal, 1: mild, 2: moderate, 3: severe, 4: severe with affection of serosa), hyperplasia of crypts (0: no, 2: yes), degree of ulceration (0: no ulcers, 1: 1–2 ulcers involving up to 20 crypts, 2: 1–4 ulcers involving 20 to 40 crypts, 3: any ulceration exceeding the aforementioned criteria), submucosal edema (0: no, 1: yes), bleeding (0: no, 1: yes) and the total area of affected tissue (0: 0%, 1: ≤ 30%, 2: ≤ 70%, 3: >70%). The maximum score thus sums up to 14. Infiltration of eosinophilic granulocytes into inflamed cola was quantified by counting the eosinophils in four high power field micrographs of submucosal edema in relation to the total number of submucosal immune cells.

### Statistical analysis

Data are represented as arithmetic mean of replicates ± 95% CI for each parameter. Group sizes were *n* = 5 (WT, +DSS), 5 (IL-5-deficient, not reconstituted, +DSS), 7 (IL-5-deficient, reconstituted, -DSS), 10 (IL-5-deficient, reconstituted, +DSS). Outliers as identified using ROUT with an aggressiveness Q of 0.1%, were removed before subsequent analyses. Statistical analyses were performed with GraphPad Prism version 6.07 for Windows (GraphPad Software, La Jolla, CA, USA) using two-way ANOVA with *post-hoc* Tukey test for evaluating significance during the repeated measurements of DAI and body weight and one-way ANOVA with *post-hoc* Holm-Sidak test for evaluating significance in all other measurements. Differences between group means were considered significant if *p* < 0.05.

## Results

**Figure 2:**
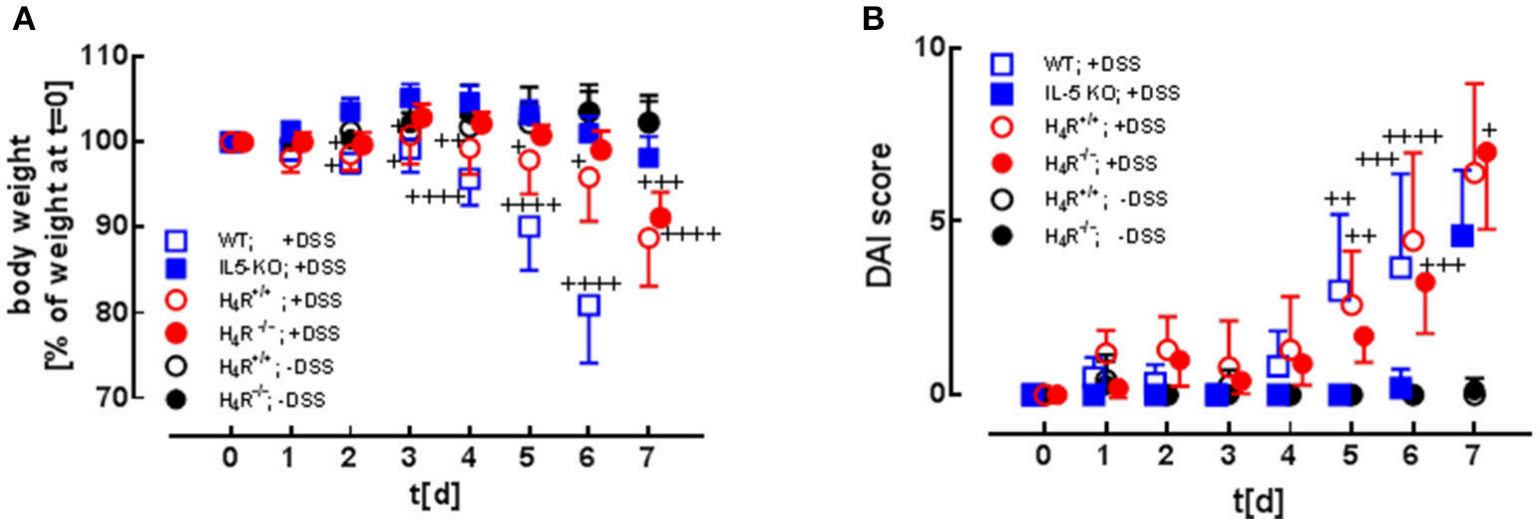
Clinical and macroscopic analyses. WT mice and IL-5-deficient mice, either left not reconstituted (IL-5 KO) or reconstituted with eosinophils obtained from IL-5-competent isogeneic wild-type (H_4_R^+/+^) or H_4_R-deficient (H_4_R^−/−^) mice, were fed with water containing DSS for 7 days. During this period, the clinical parameters body weight (**A**; due to severe symptoms DSS-fed WT mice had to be taken off the experiment at day 6), stool consistency, and perianal bleeding (not reported) were recorded and evaluated according to a scoring system. The three individual parameters are summarized to obtain the disease activity index (DAI) **(B)** [means ± 95% CI; *n* = 5 (WT +DSS), 5 (IL-5 KO, +DSS), 7 (reconstituted, -DSS), 10 (reconstituted, +DSS); 2-way ANOVA with Tukey's post-test (vs. IL-5 KO): ^+^*p* < 0.05, ^++^*p* < 0.01, ^+++^*p* < 0.001, ^++++^*p* < 0.0001].

Colitis was induced by DSS application in BALB/cJ WT mice and in BALB/cJ IL-5-deficient mice either or not reconstituted with isogenic bone marrow-derived eosinophils. The transferred eosinophils had an either H_4_R^+/+^ or an H_4_R^−/−^ genotype. For a period of 7 days after induction of colitis, the mice's body weights (Figure 2A) (16) and disease activity indices (DAI; Figure 2B) were recorded daily and calculated, respectively. In control mice, which were fed pure water without DSS, body weight increased by roughly 5% during the observation period of 7 days, while stool consistency remained normal and perianal bleedings were not observed. Thus, for control mice the DAI score was 0 for the whole observation period (Figure 2B). Compared to WT mice, lack of IL-5 expression in BALB/cJ mice significantly ameliorated DSS-induced body weight loss (Figure 2A) and delayed the onset of enhanced DAI (Figure 2B). Reconstitution of IL-5-deficient mice with eosinophils led to an aggravation of DSS-induced clinical signs of colitis (Figures 2A,B), the H_4_R^+/+^ eosinophils' transfer resulting in an earlier onset and a faster enhancement of the DAI than the H_4_R^−/−^ eosinophils' transfer. Thus, the expression of H_4_R on eosinophils affects the kinetics of clinical signs occurring in acute DSS-induced colitis in BALB/cJ mice.

**Figure 3:**
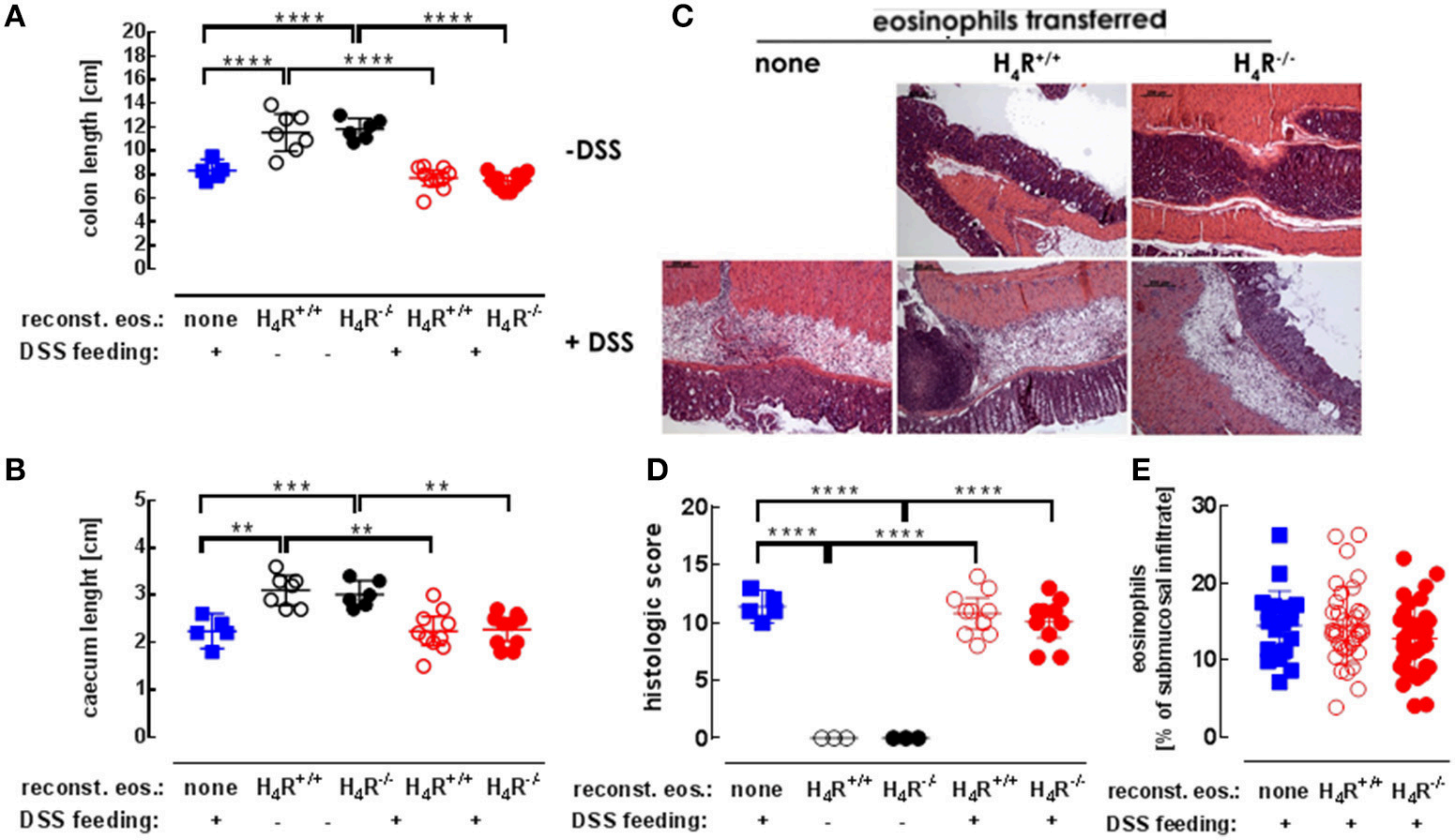
Histological analyses. IL-5-deficient mice were either left not reconstituted (none) or were reconstituted with eosinophils obtained from IL-5-competent isogeneic wild-type (H_4_R^+/+^) or H_4_R-deficient (H_4_R^−/−^) mice and then fed with water either or not containing DSS for 7 days. At day seven, the mice were dissected and the lengths of cola **(A)** and ceca **(B)** were recorded. The cola were histologically prepared, stained with H&E (**C**; exemplary photographs) and quantitatively analyzed using a defined scoring system in a blinded manner **(D)**. Eosinophils within the infiltrations were identified and quantitated using 3–4 high power field areas per colon **(E)** (single values and means ± 95% CI; 1-way ANOVA with Holm-Sidak's post-test: **p < 0.01, ***p < 0.001, ****p < 0.0001).

Morphological and histological alterations of caeca and cola are parameters indicative for colitis. Application of DSS in the drinking water significantly reduced the lengths of cola (Figure 3A) and caeca (Figure 3B) in eosinophil-reconstituted IL-5-deficient mice. Differences between DSS-treated mice either or not reconstituted with eosinophils were detected neither in caeca nor in cola. Hence, also the genotype of the transferred eosinophils did not affect the lengths' reduction (Figures 3A,B). Histological examination of the colon tissues revealed similar degrees of DSS-induced destruction in not reconstituted and in reconstituted IL-5-deficient mice, which was again not affected by the eosinophils' genotype (Figures 3C,D). All samples of DSS-treated mice show severe edema formation, crypt deformation, and inflammatory infiltrations (Figure 3C). Within these submucosal infiltrations, the percentage of eosinophils was similar in tissues obtained from not reconstituted and from reconstituted IL-5-deficient mice (Figure 3E). As observed in preliminary experiments, about 15% of the transferred eosinophils (4.5 × 10^6^; i.v. injection) could be recovered in lung, spleen, and liver 1 day after injection, the by far most of them residing in the liver. However, at the end of the 7 days observation period no transferred eosinophils could be identified any more among the eosinophils in the intestinal infiltrations.

**Figure 4:**
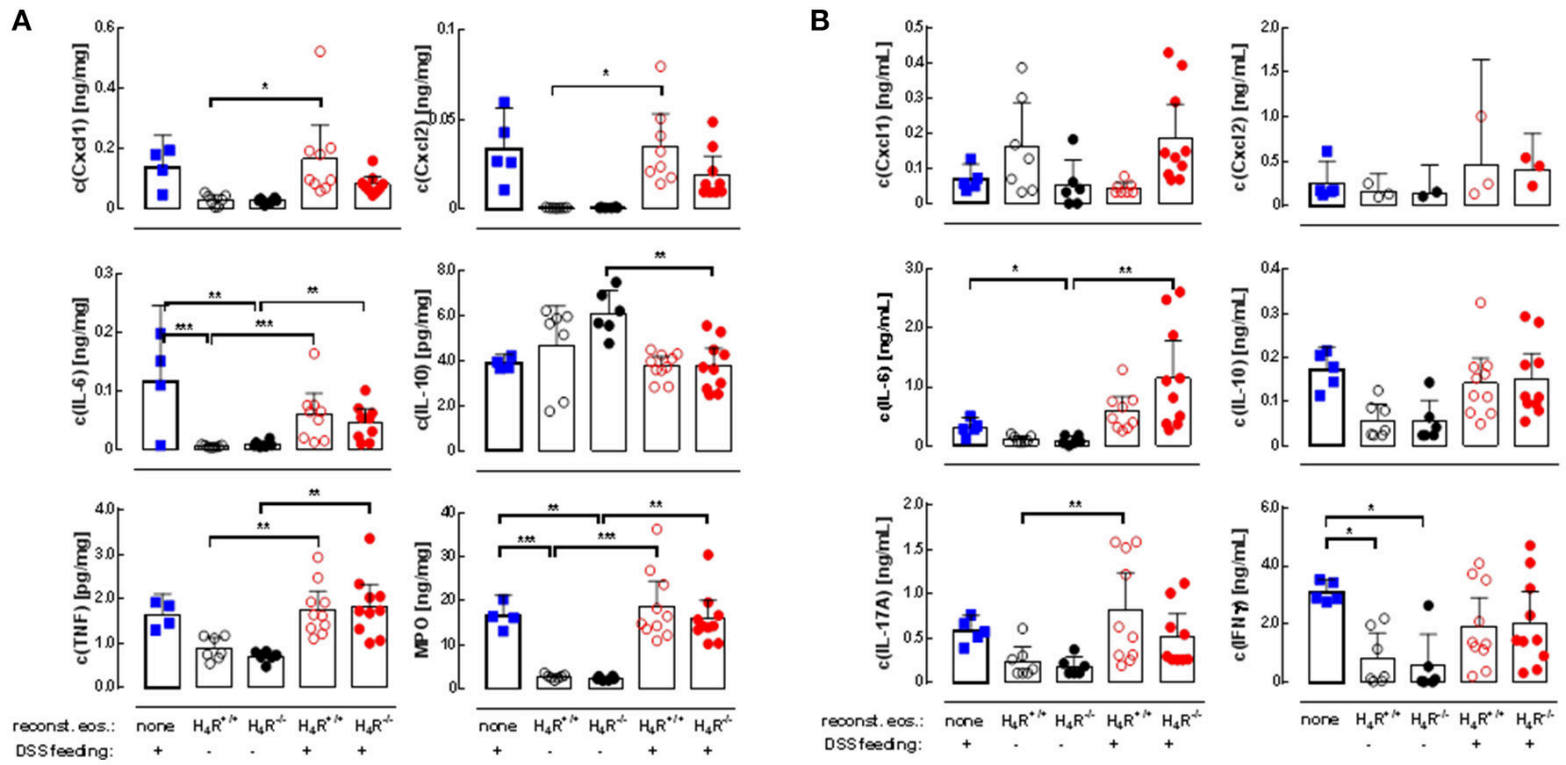
Cytokine production in cola and re-stimulated lymph node cells. IL-5-deficient mice were either left not manipulated (none) or were reconstituted with eosinophils obtained from IL-5-competent isogeneic wild-type (H_4_R^+/+^) or H_4_R-deficient (H_4_R^−/−^) mice and then fed with water either or not containing DSS for 7 days. At day seven, the mice were dissected and cola and mesenteric lymph nodes were obtained. Cola were homogenized and cleared, and lymph nodes were processes to single cells, which were stimulated *in vitro* with CD3 antibodies. Cytokine concentrations were measured in colon homogenates **(A)** and conditioned lymph node cell supernatants **(B)** by multiplex assays (single values and means ± 95% CI; outliers, identified using ROUT with an aggressiveness Q of 0.1%, were removed; 1-way ANOVA with Holm-Sidak's post-test: *p < 0.05, **p < 0.01, ***p < 0.001).

The DSS-induced inflammatory reaction in the colon was evaluated by analyzing the local mediator expression (Figure 4A). DSS feeding led to an enhanced production of CXCL1, CXCL2, IL-6, TNF, and MPO, and a reduced production of IL-10 in IL-5-deficient mice, which was not affected by reconstitution of the mice with eosinophils, independently of the genotype of the transferred cells (Figure 4A). No significant differences between DSS-fed and not DSS-fed mice were found in IL-1β, IL-12, IL-17, IL-23, and IFNγ production (Table 1A). In cells obtained from the gut-draining lymph nodes of IL-5-deficient mice, αDC3-re-stimulation resulted in only slight tendencies toward an enhanced production of the mediators IL-6, IL-10, IL-17, and IFNγ due to the previous *in vivo* DSS application (Figure 4B). For the expression of CXCL1, CXCL2, TNF, IL-1β, IL-12, and IL-23 such trends were not observed (Figure 4B, Table 1B). Thus, regarding these end-point parameters, no function can be attributed to the H_4_R on eosinophils.

**Table 1A:** Cytokine concentrations in colon tissue (pg/mg tissue; mean ± SD).

**Reconst. eos**	**DSS feeding**	**IL-1β**	**IL-12p70**	**IL-17A**	**IL-23p19**	**IFNγ**
None	+	81 ± 16	9 ± 4	< LLOQ	112 ± 26	< LLOQ
H_4_R^+/+^	–	91 ± 32	8 ± 5	< LLOQ	142 ± 70	< LLOQ
H_4_R^−/−^	–	79 ± 15	7 ± 2	< LLOQ	130 ± 24	< LLOQ
H_4_R^+/+^	+	86 ± 25	9 ± 3	< LLOQ	104 ± 21	< LLOQ
H_4_R^−/−^	+	80 ± 24	9 ± 4	< LLOQ	106 ± 33	< LLOQ

**Table 1B:** Cytokine concentrations in supernatants of re-stimulated lymph node cells (pg/ml; mean ± SD).

**Reconst. eos**	**DSS feeding**	**TNF**	**IL-1β**	**IL-12p70**	**IL-23p19**
None	+	191 ± 30	299 ± 10	< LLOQ	262 ± 53
H_4_R^+/+^	–	129 ± 51	126 ± 157	< LLOQ	180 ± 79
H_4_R^−/−^	–	102 ± 88	98 ± 152	< LLOQ	175 ± 86
H_4_R^+/+^	+	144 ± 62	236 ± 124	< LLOQ	273 ± 104
H_4_R^−/−^	+	141 ± 64	177 ± 152	< LLOQ	282 ± 120

**Figure 5:**
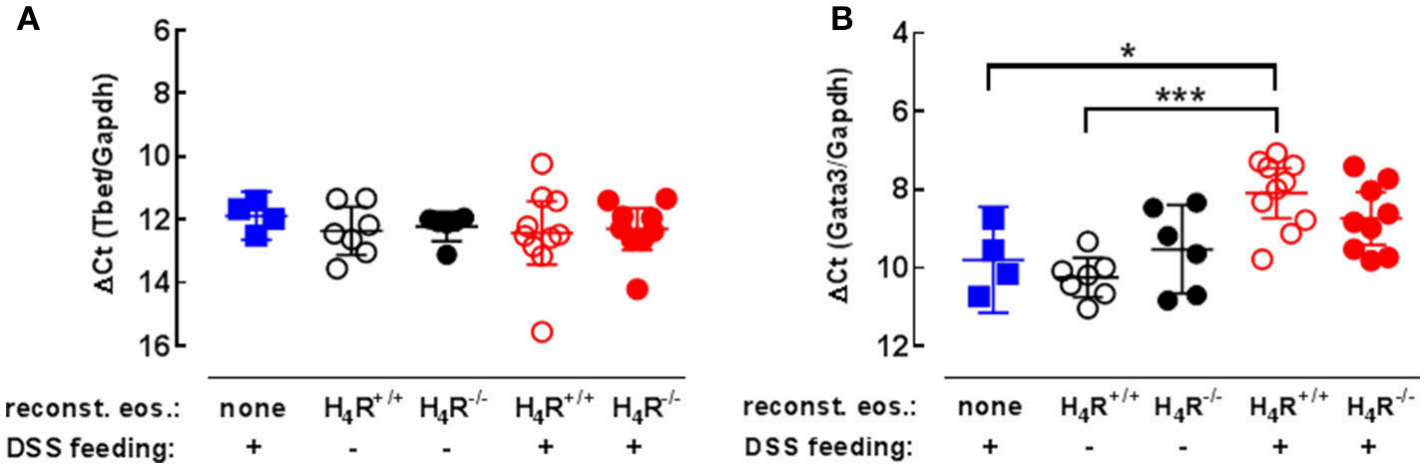
Th1/Th2-type transcription factor expression in cola. IL-5-deficient mice were either left not manipulated (none) or were reconstituted with eosinophils obtained from IL-5-competent isogeneic wild-type (H_4_R^+/+^) or H_4_R-deficient (H_4_R^−/−^) mice and then fed with water either or not containing DSS for 7 days. At day seven, the mice were dissected and cola were obtained. RNA was extracted from cola and the relative quantity of T-bet **(A)** and GATA3 **(B)** was analyzed by RT-qPCR (single values and means ± 95% CI; 1-way ANOVA with Holm-Sidak's post-test: *p < 0.05, ***p < 0.001).

Since histamine via the H_4_R is able to modulate T-cell polarization, the expressions of Th1- and Th2-specific transcription factors were measured in colonic tissues to analyze the local immune response. While the expression of Th1-specific Tbet was not affected by DSS-feeding nor by the transfer of eosinophils (Figure 5A), Th2-specific GATA3 expression was significantly enhanced due to the transfer of H_4_R^+/+^ eosinophils to IL-5-deficient mice in combination with DSS feeding, while transfer of H_4_R^−/−^ eosinophils and DSS feeding did not result in such an enhancement (Figure 5B). Thus, in contrast to H_4_R^−/−^ eosinophils, H_4_R^+/+^ eosinophils promote the infiltration of affected colon tissue by Th2 cells in BALB/cJ mice.

## Discussion

Eosinophils functionally express H_4_R, which is involved in regulation of the cells' migration and activation (4, 6, 8). However, in *in vitro* systems, histamine, as compared to other stimuli, is only a partial activator of eosinophils (8). In the present study, we aimed at analyzing the role of H_4_R on eosinophils *in vivo*, employing DSS-induced colitis in mice, a model for human UC. The pathophysiological manifestations of UC and DSS-induced colitis are caused by an underlying inflammatory immune response, which essentially depends on the presence of eosinophils and their products (25–27).

### IL-5 is a pathogenic factor in DSS-induced acute colitis in BALB/cJ mice

Differentiation of eosinophils from progenitor cells in the bone marrow is driven by IL-5 in concert with IL-3 and GM-CSF. Moreover, IL-5 stimulates the newly generated eosinophils' mobilization and promotes their eotaxin-mediated recruitment to the gastrointestinal tract (19, 20). Although in mice lacking IL-5 expression the infection-induced enhancement of eosinophil numbers is abolished (21), IL-5 deficiency is reported to be without effect on the pathogenesis of acute DSS-induced colitis (13, 26). These latter studies, however, were performed using mice on a C57Bl/6 background, which differ in their susceptibility to induction of acute colitis by DSS as compared to BALB/cJ mice (28, 29). Moreover, in contrast to C57Bl/6 mice, BALB/cJ mice are prone to preferentially mount a Th2-type immune response, and Th2 cells are the main T cell subset found in inflammatory infiltrations in both human UC and DSS-induced colitis in mice (30–32). Consequently, in the present study we observed that lack of IL-5 expression in BALB/cJ mice delays the appearance of typical clinical symptoms. Hence, the disease-aggravating effect of IL-5 on acute DSS-induced colitis indeed depends on the genetic background of the mouse model employed. Such marked strain-dependent differences between BALB/c and C57Bl/6 mice have already been observed in other experimental models (33). Lastly, one has to keep in mind that none of the different animal model fully resembles human IBD but only some specific features. Hence, DSS-feeding induces a pathology representing the acute inflammation occurring in UC, while TNBS administration more closely models CD. Moreover, the genotype of a given individual, mouse or man, can affect the degree of polarization of an occurring immune response. Thus, caution has to be paid when aiming at transferring results obtained using a congenic strain of mice to the genetically inhomogeneous human population.

### The pathogenic effect of eosinophils in acute DSS-induced colitis is promoted by the H_4_R

The delayed early kinetic of DSS-induced colitis symptoms in IL-5-deficient mice is reversed by reconstituting the mice with H_4_R^+/+^ eosinophils. Thus, this model provides a reliable *in vivo* system to analyze the contribution of eosinophils.

The capacity of the transferred eosinophils to revert the clinical phenotype of DSS-treated IL-5-deficient mice is affected to only a minor extent by the H_4_R genotype of the donor mice. Moreover, the effect of the eosinophils' transfer on the signs of colitis is only transient, being virtually absent at day 7 after colitis induction. This transient kinetic and the rather small difference between the effects of reconstituting eosinophils obtained from H_4_R^+/+^ or H_4_R^−/−^ mice in this model indicates that also *in vivo* histamine via the H_4_R only partially activates eosinophils, which is in accordance with the corresponding *in vitro* observations (8).

### H_4_R on eosinophils shifts the local immune response toward the Th2 type

Only the transfer of eosinophils obtained from H_4_R^+/+^ mice, but not that from H_4_R^−/−^ mice results in a significantly enhanced GATA3 expression in the inflamed colon. Similar observations were also made in biopsies obtained from patients suffering from active UC, in which GATA3 expression correlated with disease activity, and in mice with colitis induced by oxazolone treatment (34). GATA3 is of relevance for the differentiation of breast and urogenital epithelial tissues and of Th2 cells, but it is virtually absent in respiratory and gastrointestinal epithelia (35). Moreover, overexpression of GATA3 in T cells accelerates colitis induced in mice by DSS feeding (36). Thus, the enhanced expression of GATA3 observed in the present colitis study indicates a local immune response biased toward the Th2-type which is supported by the H_4_R genotype of the transferred eosinophils. Such an effect of the H_4_R on polarization of an immune response has so far been ascribed only to dendritic cells, in which the H_4_R is involved in the regulation of cytokine and chemokine production (37–41). The precise mechanism, however, by which mechanism the H_4_R on eosinophils affects the polarization of the immune response in DSS-induced colitis in BALB/cJ mice still has to be elucidated.

**Figure 6:**
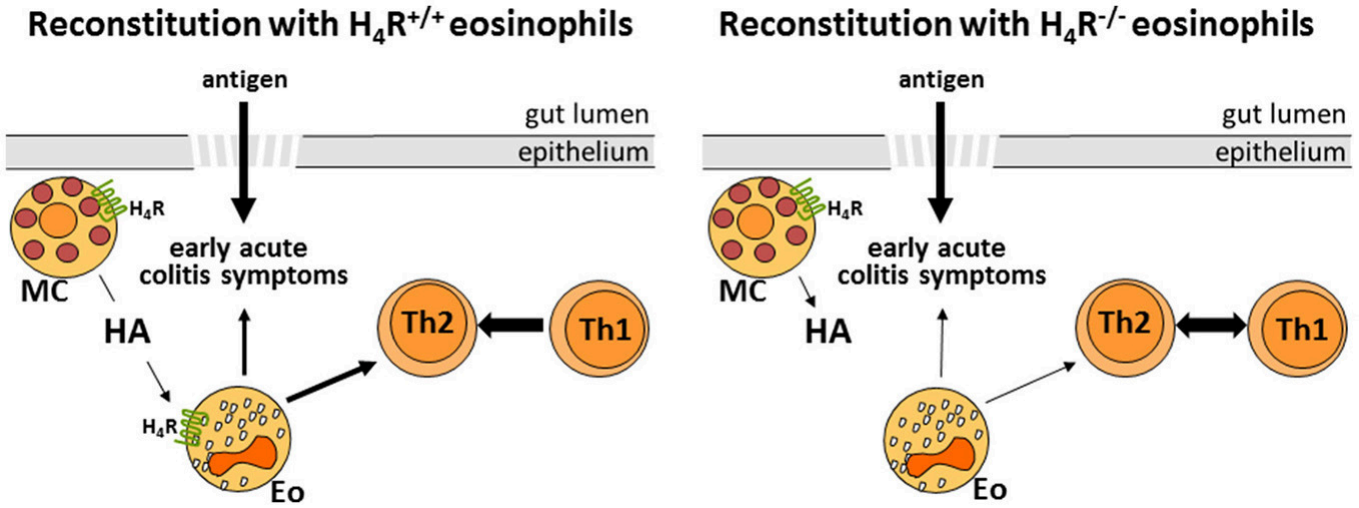
Schematic representation of the H_4_R function on eosinophils in experimental colitis (HA, histamine; MC, mast cell; Eo, eosinophil; Th1/Th2, T helper 1/2 cell).

In conclusion (Figure 6), this study indicates that, as observed already *in vitro*, also *in vivo*, in the mouse model of DSS-induced colitis, histamine via the H_4_R only partially activates eosinophils with respect to the acute inflammatory reaction. Here, probably other innate immune cells, such as mast cells provide the major H_4_R contribution (42). On the initiating adaptive immune response, however, H_4_R on eosinophils seem to provide a regulatory function.

## Author contributions

BS and DN designed the study. BS and LB performed the experiments. BS and DN interpreted the data. BS, DN, and RS drafted and/or critically revised the manuscript. BS, DN, LB, and RS approved publication of the content. BS, DN, LB, and RS agree to be accountable for the content.

### Conflict of interest statement

The authors declare that the research was conducted in the absence of any commercial or financial relationships that could be construed as a potential conflict of interest.
